# Incidence, prevalence, and natural history of primary sclerosing cholangitis in the United Kingdom

**DOI:** 10.1097/MD.0000000000007116

**Published:** 2017-06-16

**Authors:** Huifang Liang, Sudhakar Manne, Jesse Shick, Trevor Lissoos, Paul Dolin

**Affiliations:** a Takeda Development Center Americas, Inc.; b Takeda Pharmaceuticals U.S.A., Inc., Deerfield, IL; c Takeda Development Centre Europe Ltd., London, UK.

**Keywords:** Clinical Practice Research Datalink (CPRD), hazard ratio, incidence, natural history, prevalence, primary sclerosing cholangitis (PSC)

## Abstract

Primary sclerosing cholangitis (PSC) is a rare obliterative fibrotic condition of the bile ducts. We assessed PSC epidemiology and natural history within the UK Clinical Practice Research Datalink (CPRD).

Incidence and natural history of PSC were evaluated in a retrospective cohort study using linkage of CPRD, Hospital Episode Statistics, and Office for National Statistics data. Data from age, sex, and general practice-matched population controls provided a context for the incident PSC patients. Liver disease other than PSC was defined as autoimmune hepatitis, hepatitis, hepatomegaly, liver failure, cirrhosis, portal hypertension, cholangiocarcinoma, or hepatobiliary cancer.

The age-standardized incidence of PSC was 0.68 (95% confidence interval [CI] 0.45–0.99) per 100,000 person-years and the age-standardized prevalence was 5.58 (95% CI 4.82–7.35) per 100,000 during 1998 to 2014. In all, 250 incident PSC patients met the inclusion criteria and each was matched with 5 controls (mean age 54 ± 18 years, men 63.2%). A higher percentage of PSC patients had a history of inflammatory bowel disease (54% vs 2%) and liver disease other than PSC (22% vs 1%) than controls (standardized difference_weighted_ >0.1). During a median follow-up of 5 years, PSC patients were more likely to develop adverse health outcomes. The mortality rate per 1000 person-years was 3-fold higher in PSC than population controls (49.5 vs 16.1; incidence rate ratio 3.1, 95% CI 2.2–4.2).

The incidence and prevalence of PSC observed in the UK CPRD were either comparable with or higher than previous studies. Compared with the general population, PSC patients had worse health outcomes including PSC disease progression, complications, and higher mortality.

## 1 Introduction

Primary sclerosing cholangitis (PSC) is a rare disease of the bile ducts, characterized by ongoing inflammation, obliteration, and fibrosis of both intrahepatic and extrahepatic bile ducts.^[1]^ Patients with PSC, often found in association with inflammatory bowel disease (IBD),^[2–4]^ are at increased risk of cholangiocarcinoma, hepatobiliary cancer, and colorectal cancers.^[5,6]^ Understanding PSC patient demographics, medical histories, liver functions, hematology tests, and disease progression may help with disease management. PSC epidemiological studies to date have varied in sample size,^[1,7]^ and data from nationally representative samples are limited.^[8]^ The objective of this study is to describe the epidemiology of PSC, baseline characteristics of patients before diagnosis, and the course of the disease after diagnosis.

## 2 Patients and methods

### 2.1 Data source

The UK Clinical Practice Research Datalink (CPRD) is an electronic medical record database that has been available since 1987 and covers 7% of the UK population.^[9]^ The subset of the CPRD with linkage of primary care medical and prescribing records with hospital in-patient morbidity and procedures data, hospital out-patient morbidity data (a.k.a. Hospital Episode Statistics [HES]), and Office for National Statistics (ONS) mortality data was used for this study. The validity and representativeness of the CPRD dataset has been described elsewhere.^[9]^

### 2.2 Study population

The population included all patients who were registered at any time during 1998 to 2014 with a general practice (GP) in the CPRD linkage subset where the GP practice's data were classified by CPRD as “up to research standard.”

Incident PSC was defined as having a new diagnosis of PSC, based on Read code = J661700, without a secondary cause of sclerosing cholangitis at any time during 1998 to 2014. Incident cases were also required to have at least 1 year of registration time in the CPRD before the PSC diagnosis to differentiate between prevalent and incident cases of PSC as previously demonstrated by Lewis et al.^[10]^

A general population comparator group was drawn from the CPRD linkage subset. For each incident PSC patient, up to 5 population controls were selected matched on year of birth, sex, and GP practice. Patients with PSC diagnosis at any time were not eligible for inclusion in the comparator group. The PSC diagnosis date of each incident PSC case was used as a pseudo-diagnosis date for the matched controls.

All data in the CPRD linkage subset were de-identified before release for research. The study protocol was approved by the CPRD Independent Scientific Advisory Committee (ISAC) (ISAC protocol number CPRD15_139A) for Medicines & Healthcare products Regulatory Agency (MHRA) Database Research before study conduct.

### 2.3 Variables of interest

The natural history outcomes consisted of those clinically associated with PSC, including development of IBD, cholangitis, bile duct strictures, gallstones, liver failure, and need for liver transplantation, and complications of PSC, including cirrhosis, portal hypertension, cholangiocarcinoma, hepatobiliary cancer, colorectal cancer, pancreatic cancer, and death. Medical histories were assessed for these conditions using all available data to differentiate between pre-existing morbidity and incident outcomes occurring after PSC diagnosis.

Patient characteristic variables included demographics (age at index date, sex, year of diagnosis), duration of CPRD registration before diagnosis date, duration of follow-up after diagnosis, and family history of cancer. In addition, baseline laboratory values were analyzed using the most recent test results within 12 months before diagnosis date. Elevated values for alanine aminotransferase (ALT), aspartate aminotransferase (AST), alkaline phosphatase (ALP), and gamma-glutamyl transpeptidase (GGT) were defined as >1× and >3× upper limit normal [ULN], with the ULN value being 20 IU/L for ALT, 20 IU/L for AST, 120 U/L for ALP, and 42 IU/L for GGT.^[11]^ Elevated total bilirubin was defined as >1.5 × ULN (1.5 mg/dL); low serum albumin was defined as <3.5 g/dL. Anemia was defined as hemoglobin <13 mg/dL in men and <12 mg/dL in women.^[12]^ Low white blood cell (WBC) count was defined as WBC <4.5 × 10^9^/L, and high WBC count was defined as WBC >11 × 10^9^/L. Thrombocytopenia was defined as platelet count <150 × 10^9^/L. Elevated C-reactive protein (CRP) was defined as CRP ≥2.1 mg/L. Elevated prothrombin time (PTT) was defined as >15.2 seconds, and elevated international normalized ratio (INR) defined as >2.^[12]^

### 2.4 Statistical analysis

The crude incidence rate of PSC during 1998 to 2014 combined and annually, stratified by age group and sex, was estimated using the total number of new cases of PSC diagnosed as the numerator and the cumulative PSC-free person-years as the denominator during 1998 to 2014 and in each year. Age and/or sex-standardized rates and 95% confidence intervals (CIs) were calculated for both sexes combined and for each sex via direct standardization using the age and/or sex-specific UK population estimate in mid-2013 as the reference population.^[13]^ To minimize random variation, incidence rates for 3-year moving averages were calculated.^[14]^ The period prevalence of PSC between 1998 and 2014, and point prevalence at mid-year (July 1) of 1998, 2002, 2006, 2010, and 2014 were estimated using total number of PSC patients (prevalent and incident) as the numerator and the total number of patients in CPRD as the denominator for the period of 1998 to 2014 and at each time point, respectively. Office Excel 2007 (Microsoft Corporation, Seattle, WA) was used to generate line graphs for prevalence and incidence.

Descriptive statistics were generated for baseline characteristics for PSC patients and matched general population, with weighted standardized difference of 0.1 used as a threshold to indicate group difference for demographics and disease histories.^[15,16]^ Inferential statistics (independent *t* test and chi-square test) were used to compare liver function tests and hematological tests between PSC and non-PSC patients. The incidence rates for the natural history outcomes were calculated by dividing the number of patients with the outcomes by the total number of person-years of follow-up. The follow-up time for each outcome began from PSC diagnosis date and ended on the earliest of any of the following censoring events: the date of the incident natural history outcome; transfer out date of the GP; death date; GP record end date; and end of study period (December31, 2014). Incidence rates ratios (IRRs) and 95% CIs were calculated, and Kaplan–Meier survival curves for natural history outcomes were generated for incident PSC cases and general population, and log-rank tests were used to assess statistical significances. Data management and analyses were performed in SAS 9.4 (SAS Institute Inc., Cary, NC). To protect patient confidentiality, all data presentations with less than 5 events in a stratum have been suppressed.

## 3 Results

### 3.1 Incidence of PSC

**Figure 1:**
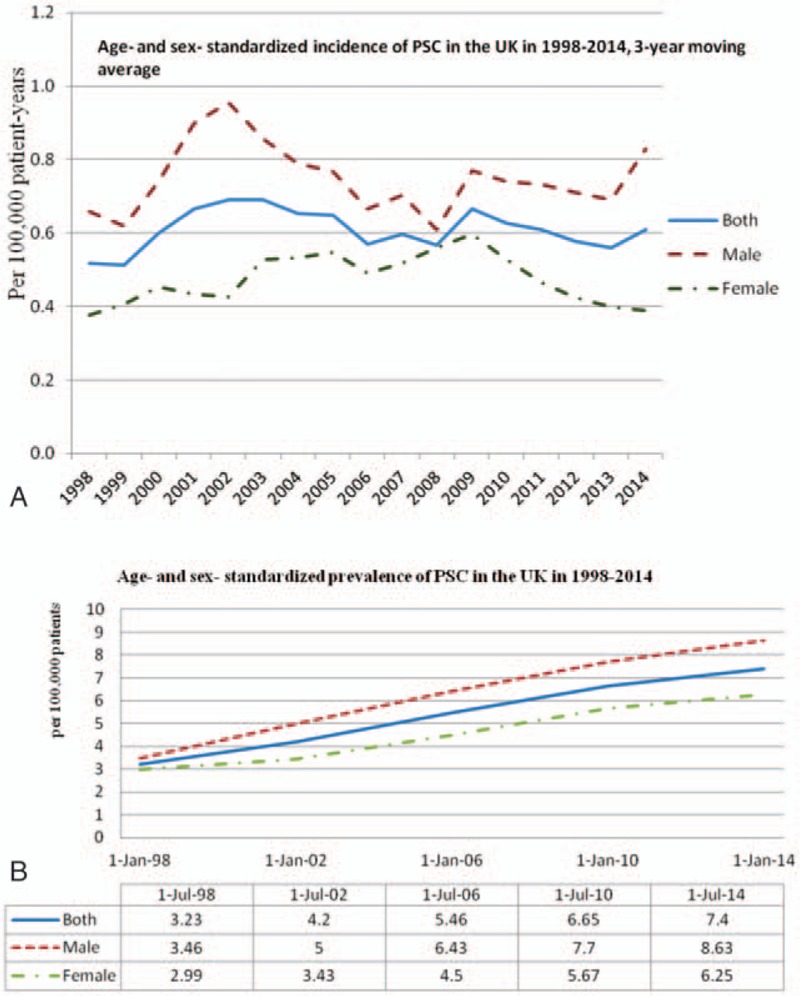
(A) Age and sex-standardized incidence of PSC in the UK in 1998 to 2014. (B) Age and sex-standardized prevalence of PSC in the UK in 1998 to 2014. PSC = primary sclerosing cholangitis.

In all, 421 incident cases of PSC occurred (255 males, 166 females; 330 with HES linkage, 121 without linkage) in 1998 to 2014. The crude incidence was 0.64 (95 CI% 0.58–0.70) per 100,000 person-years, and significantly higher in men (0.78, 95% CI 0.69–0.88) than in women (0.50, 95% CI 0.43–0.58). However, the age and sex-standardized PSC incidence rate was 0.68 (95% CI 0.45–0.99) per 100,000 person-years in 1998 to 2014, with no difference between men and women (men 0.84, 95% CI 0.56–1.18 vs women 0.52, 95% CI 0.34–0.81). The age-standardized annual incidence rate ranged from 0.51 to 0.69 per 100,000 person-years, with no clear trend across time (*P* = .93) (Fig. 1A).

### 3.2 Prevalence of PSC

There were 837 prevalent cases of PSC (500 males, 337 females) in 1998 to 2014. Crude prevalence of PSC was 6.12 (95 CI% 5.72–6.54) per 100,000 persons, and statistically higher in men than in women (men 7.58, 95% CI 6.95–8.28; women 4.75, 95% CI 4.27–5.29 per 100,000 persons). The age and sex-standardized PSC prevalence was 5.58 (95% CI 4.82–7.35) per 100,000 persons in both sexes, 6.73 (95% CI 5.28–6.68) per 100,000 persons in men, and 4.44 (95% CI 3.32–6.06) per 100,000 persons in women. Whereas there was no clear trend across time for incidence, prevalence increased from 3.23 per 100,000 patients in 1998 to 7.40 per 100,000 patients in 2014 (Fig. 1B).

### 3.3 Patient baseline characteristics

**Figure 2:**
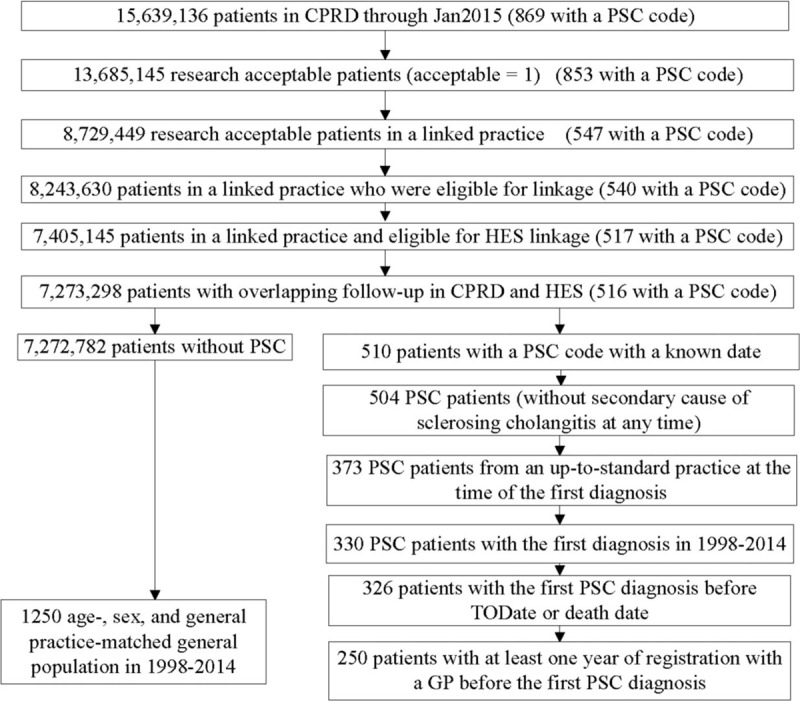
Methodology to identify incident PSC patients and matched general population in 1998 to 2014. Note: Two inclusion criteria, the index date was before the censor date and 1 year of registration with a GP before the index date, for non-PSC controls were applied in matching. PSC = primary sclerosing cholangitis.

Of 869 patients with PSC code, 250 fulfilled the inclusion criteria for incident PSC and each was matched with 5 patients from the general population drawn from the CPRD linkage subset (Fig. 2). Table 1 presents demographic and medical history, and Table 2 presents laboratory values at baseline. PSC patients were mostly male. Their mean age at diagnosis was 54 years (range 6–93), and 4.4% were aged less than 18 years.

**Table 1:**
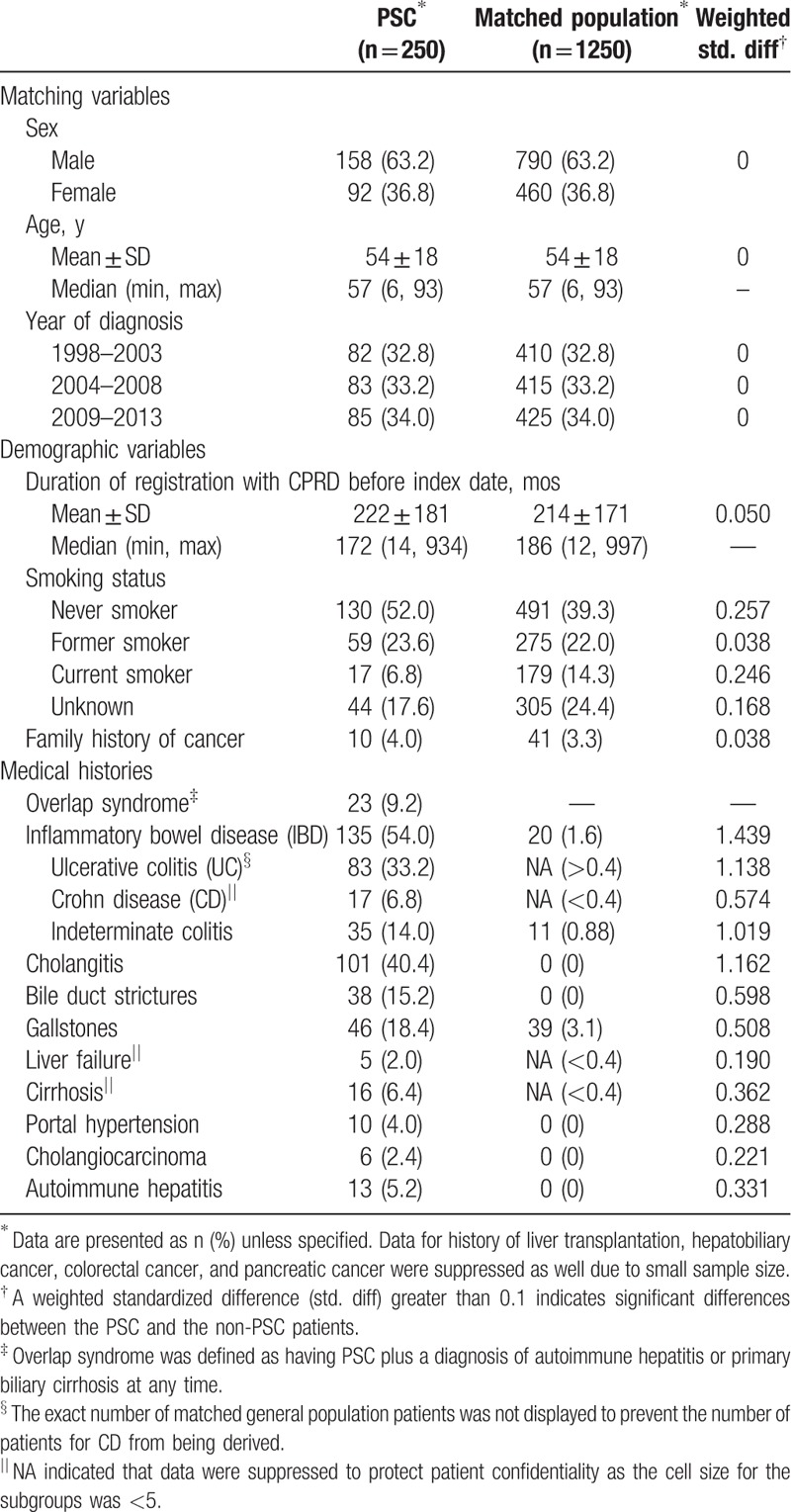
Baseline characteristics of incident primary sclerosing cholangitis patients and their age, sex, and general practice-matched general population in 1998 to 2014 in the Clinical Practice Research Datalink.

**Table 2:**
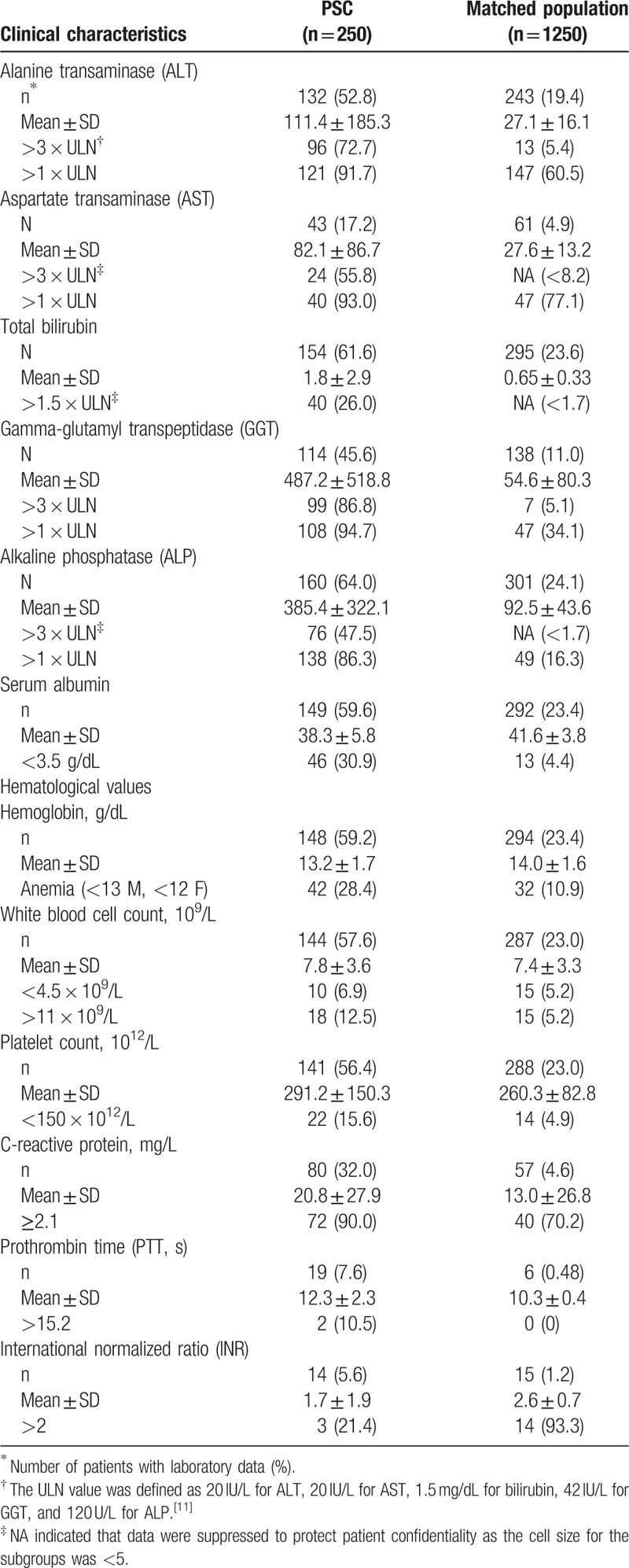
Baseline clinical characteristics of incident primary sclerosing cholangitis (PSC) patients and matched population.

Pre-existing IBD was present in 54.0% PSC cases compared with only 1.6% of the matched population, and UC was the dominant type. PSC patients with IBD were significantly younger (mean age of 50.3 ± 17.8 years) than PSC patients without IBD (mean age 57.3 ± 17.9 years; *P* = .0022). PSC patients were also more likely to have a history of other hepatobiliary morbidities than the general population.

The PSC patients were also more likely to have a liver function test or a hematological test (66.0% and 65.6%, respectively) during the 12 months before diagnosis than the general population (24.4% and 30.2%, respectively). Of these, 95.8% and 69.5% of PSC patients and 70.8% and 28.4% of the general population had an abnormal liver function test or an abnormal hematological laboratory value, respectively. Among PSC patients with liver function test results recorded, the proportions with abnormal liver enzymes (greater than 3 × ULN) were 72.7% for ALT, 55.8% for AST, 86.8% for GGT, and 47.5% for ALP. Among matched patients with liver function test results recorded, the proportions with abnormal liver enzymes (greater than 3 × ULN) were 5.4% for ALT, <8.2% for AST, 5.1% for GGT, and <1.7% for ALP for a threshold of greater than ×3 ULN. The proportion of patients with abnormal bilirubin was much higher in PSC than in matched patients (26.0% vs <1.7%).

Of patients with hematological test results, the proportion of patients with anemia, low platelet count, and high WBC count was 28.4%, 15.6%, and 12.5% in patients with PSC, but only 10.9%, 5.2%, and 4.9% in the matched population (*P* < .01). Anemia was more common in those with a history of IBD than those without IBD (40.0% vs 9.9%; *P* = .002) in the matched population, whereas in the PSC patients, history of anemia was similar among those with and without IBD (27.2% and 29.9%, respectively; *P* = .72). Instead, it was observed that among PSC patients, anemia was more frequent in those with a history of liver disease than those without liver disease other than PSC (50.0% and 21.4%, respectively; *P* = .0009). Low serum albumin was more common in PSC patients with a history of liver disease other than PSC than those without (59.5% and 21.4%, respectively; *P* < .0001), which may be indicative of liver failure, cirrhosis, or chronic hepatitis. Conversely, in the matched population, neither anemia nor low serum albumin differed between those patients with a history of liver disease and those without (both *P* > .05).

### 3.4 Natural history outcomes

The PSC patients and matched population patients were longitudinally followed up after diagnosis/pseudo-diagnosis date for a mean of 61 months and 71 months, respectively, yielding a cumulative follow-up of 1271.9 person-years and 7409.0 person-years, respectively.

Table 3 shows the incidence of commonly seen PSC natural history endpoints during follow-up. Among PSC disease progression endpoints, cholangitis had the highest incidence (162.8 per 1000 person-years), followed by IBD (61.0 per 1000 person-years) and bile duct strictures (25.3 per 1000 person-years); among PSC complication endpoints except death, those with the highest incidence rate were cirrhosis (18.6 per 1000 person-years), portal hypertension (14.5 per 1000 person-years), hepatobiliary cancer (8.8 per 1000 person-years), and cholangiocarcinoma (8.1 per 1000 person-years). Compared with the matched population, all IRRs were statistically significant except for colorectal cancer and pancreatic cancer, which had 95% CI across 1.

**Table 3:**
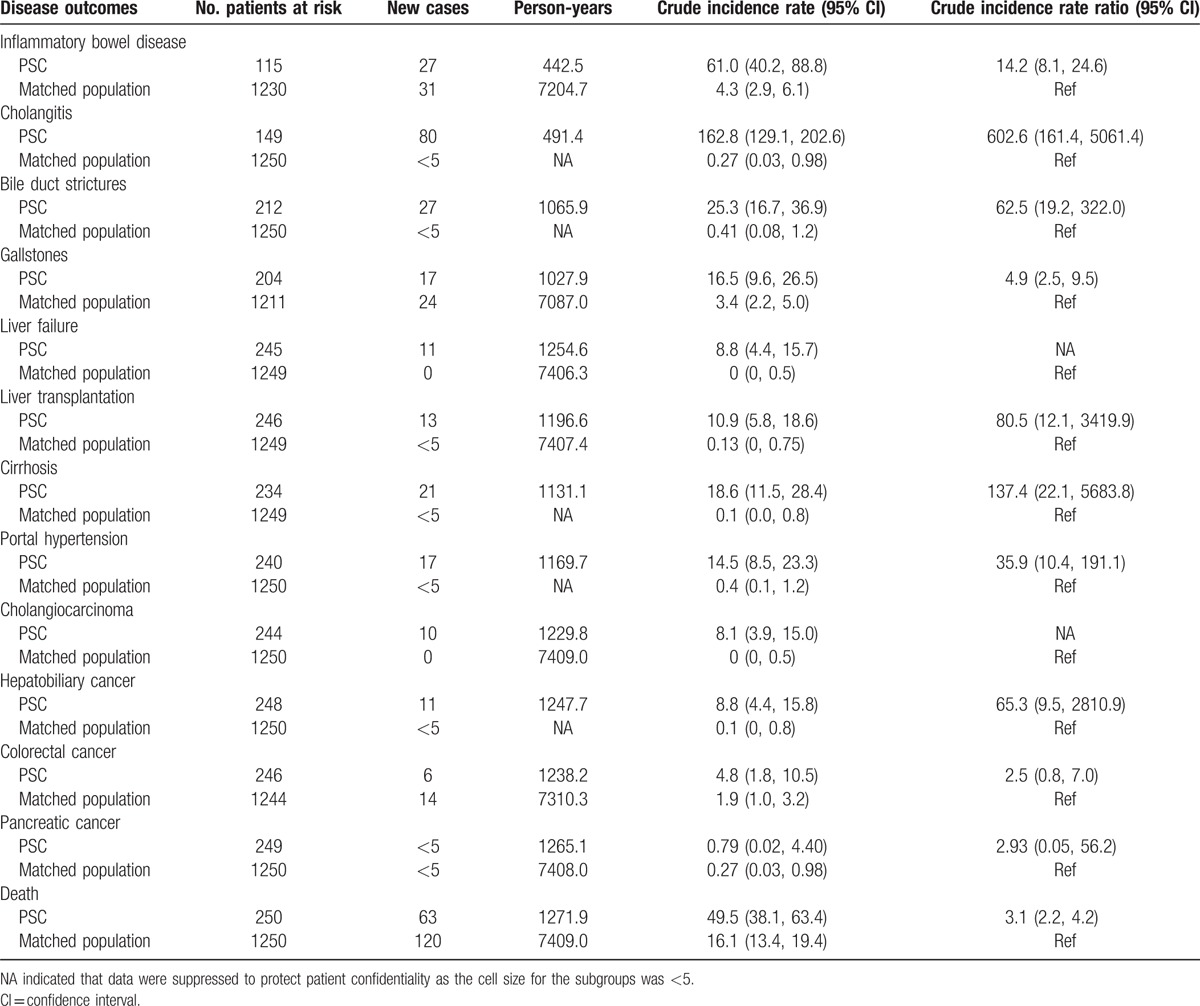
Crude incidence rate and rate ratio of disease outcomes in the primary sclerosing cholangitis (PSC) patients and matched general population (per 1000 person-years).

### 3.5 Mortality

**Figure 3:**
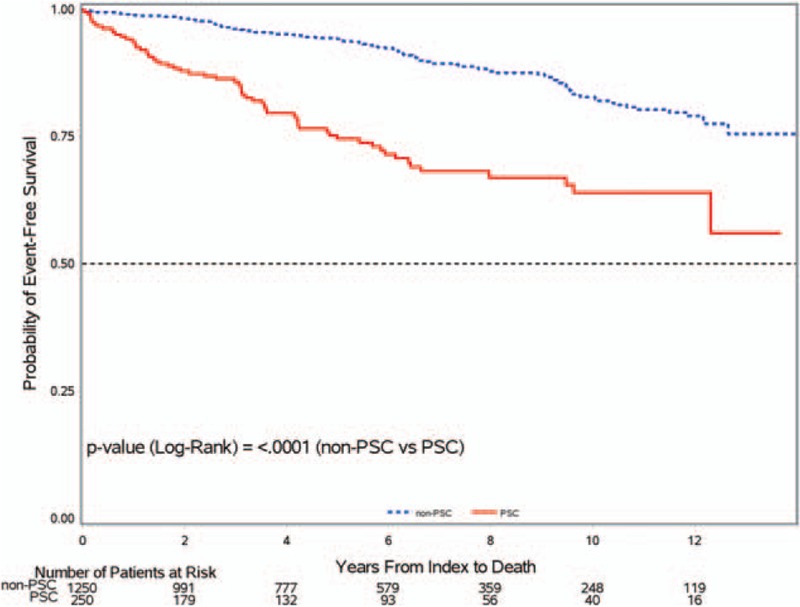
Kaplan–Meier survival curve of incident PSC patients and matched general population. PSC = primary sclerosing cholangitis.

In all, 63 of the 250 incident PSC patients died during the follow-up period, yielding a mortality rate of 49.5 per 1000 person-years. This was 3-fold higher than the mortality rate in the age, sex, and GP-matched population (IRR 3.1, 95% CI 2.2–4.2) (Table 3). Among those who died, the age at PSC diagnosis/pseudo-diagnosis was younger for PSC patients (mean = 65.1 years, SD = ±14.1) than the matched population (mean = 69.7, SD = ±10.2), and time from diagnosis/pseudo-diagnosis date to death was shorter for PSC patients (mean survival = 3.1years, SD = ±2.7) than the matched population (mean survival = 5.4 years, SD = 3.3). Figure 3 shows survival curves for PSC patients and the matched population. The observed 5-year and 10-year survival of the PSC patients was 75% and 64%, compared with 94% and 83% for the age, sex, and GP-matched population (*P* < .001).

Of the 63 deaths among PSC patients, the most frequent underlying cause of death was cancer (n = 19, 30.2%), including intrahepatic bile duct carcinoma (n = 6, 9.5%), liver or pancreatic cancer (n = 5, 7.9%), and cancers of other sites (n = 8, 12.7%). Other underlying causes of death included PSC (n = 18, 28.6%), infections (n = 7, 11.1%), PSC comorbidities such as IBD and liver disease other than PSC (n = 7, 11.1%), and heart disease and cerebrovascular disease (n = 6, 9.5%).

## 4 Discussion

This study found the age and sex-standardized incidence was 0.68 (95% CI 0.45–0.99) per 100,000 person-years, and the age and sex-standardized prevalence was 5.58 (95% CI 4.82–7.35) per 100,000 in 1998 to 2014 in the UK CPRD. To the best of our knowledge, this study of 421 incident cases and 837 prevalent cases is the largest population-based study of the epidemiology of PSC.

The incidence of PSC in this study was similar to that reported by a meta-analysis of 8 studies (incidence 0.77 per 100,000 person-years, 95% CI 0.45–1.09).^[7]^ However, of the 8 studies, only 2 had more than 100 PSC patients.^[17,18]^ One of the larger studies involved adult patients only.^[17]^ An earlier study of PSC in the UK also used the CPRD database and reported a crude PSC incidence of 0.41 per 100,000 person-years in the UK, based on 149 incident cases during 1990 to 2001.^[18]^ The PSC patients in our study and in the earlier CPRD study had similar age and sex distribution and proportion of smokers.^[18]^ Unlike the earlier study, we have standardized incidence rates to allow for temporal changes in underlying population structure in the UK, plus the current study includes an additional decade of more recent data. It is not clear why the crude incidence rate found in our study (0.64 per 100,000 person-years, 95 CI% 0.58–0.70) was higher than in the earlier study (0.41 per 100,000 per year, 95% CI 0.34–0.48). There was no temporal trend of increasing incidence with calendar year. The most likely explanation is that the present study estimated the incidence rate based on the incident PSC cases and person-years at risk (the person-year at risk for a patient who developed PSC in the middle of the year was less than a year). Conversely, the previous study used the mid-year population numbers as the denominators for that year. Each patient was considered to contribute 1 year to the denominator, which could result in an overestimate of the denominator. The previous study did not seem to exclude patients with a secondary cause of cholangitis in the PSC case definition. In addition, whether they required patient-level data that were acceptable and whether patients selected came from GPs that were considered “up to standard” were unknown. Nevertheless, both studies are consistent in showing the incidence of PSC to be very low and below 1 case per 100,000 person-years in the UK. Estimates of PSC prevalence for the overlapping years for the 2 UK studies are similar.^[18]^

A history of IBD was associated with increased occurrence of anemia in the general population, which is consistent with IBD as a known cause of anemia.^[19]^ Conversely, among PSC patients, we did not identify a relationship between IBD and anemia. Instead, we observed that anemia was associated with a history of liver disease other than PSC. As liver disorders become more advanced, they are commonly associated with anemia.^[20]^ Patients with PSC and a history of liver disease had a higher percentage of low serum albumin, which may reflect a more severe stage of their liver disease such as advanced cirrhosis or liver failure. Among those who had liver function tests, a much higher percentage of patients with PSC had elevated ALP, bilirubin, ALT and AST than in the matched population. The proportion of PSC patients with elevated ALP levels (greater than 1 × ULN) was similar to the percentage (90.8%) reported in the literature.^[21]^

Our study found a 75% 5-year survival and 64% 10-year survival for PSC patients, which was significantly worse than for the age-sex matched population. This survival rate was similar to the 64% 10-year survival rate found by Farrant et al^[22]^ in their study of the natural history of PSC in patients referred to King's College Hospital in London and to the 65% 10-year survival rate reported in a US cohort of PSC patients.^[23]^ The likely higher mortality rate in PSC patients was due to a higher rate of malignancy, liver failure, infections, and PSC comorbidities. In our study, 10 PSC patients developed a cholangiocarcinoma, whereas none occurred in the matched population group. We also found that PSC patients had a higher incidence of hepatobiliary and colorectal cancer than the matched population. Having PSC itself was the second most common cause of death in PSC patients. Although the natural history outcomes in PSC patients are well-defined in the literature,^[8,22]^ we have, for the first time, quantified the risk in a population-representative sample of PSC patients.

### 4.1 Strengths and limitations

There are several strengths to this study. Firstly, to the best of our knowledge, this study has the largest PSC patient sample size and the most recent data to determine the incidence and prevalence of PSC, compared with prior UK population studies.^[7,18]^ Additionally, secondary causes of cholangitis were excluded from the analysis, so the PSC definition is accurate. Secondly, linkage of hospital and national mortality data to CPRD primary care records was used to assess medical histories and disease outcomes. In addition to using linkage to capture events diagnosed in hospitals, but not recorded in primary care records, this study included a 1-year minimum baseline period to differentiate between incidence and prevalence of PSC, and had a sufficiently long follow-up period to enable us to assess PSC outcomes. Finally, this study included an age, sex, and GP-matched general population group of patients to provide context to the observed incidence rates.

The study is subject to a few limitations. First, patients with PSC could be under-reported or misclassified using the CPRD Read diagnostic code alone. In the UK, PSC is more likely to be managed by a specialist than a primary care physician. Therefore, PSC may be under-recorded in primary care records. Second, potential biases are possible. Some laboratory data may not be completely recorded in the database. If laboratory values are recorded in a CPRD comments field, or stored as a pdf attachment, then such information will be missing for the laboratory value data fields available in the research dataset. In addition, imbalance in smoking at baseline (more “never smokers” and fewer “current smokers” in PSC patients) might bias the results toward the null. Third, there is a possibility of unmeasured confounders that could have influenced mortality outcome in the study.

## 5 Conclusions

The incidence and prevalence of PSC observed in the UK CPRD were comparable with or higher than previous studies.

Compared with the general population, incident PSC patients had a high incidence of IBD, cholangitis, gallstones, liver transplantation, cirrhosis, portal hypertension, cholangiocarcinoma, and death. We quantified the risks of these endpoints in nationally representative data. Findings from this study may benefit clinicians who manage PSC patients in their clinical practice.
